# Bringing Light into the Dark—Overview of Environmental Impacts of Carbon Fiber Production and Potential Levers for Reduction

**DOI:** 10.3390/polym16010012

**Published:** 2023-12-19

**Authors:** Tobias Manuel Prenzel, Andrea Hohmann, Tim Prescher, Kerstin Angerer, Daniel Wehner, Robert Ilg, Tjark von Reden, Klaus Drechsler, Stefan Albrecht

**Affiliations:** 1 Department Life Cycle Engineering GaBi, Fraunhofer Institute for Building Physics IBP, Nobelstrasse 12, 70569 Stuttgart, Germany; 2 Fraunhofer Institute for Casting, Composite and Processing Technology IGCV, Am Technologiezentrum 2, 86159 Augsburg, Germany; 3 Institute for Acoustics and Building Physics IABP, University of Stuttgart, Pfaffenwaldring 7, 70569 Stuttgart, Germany; 4 Composites United e.V., Oranienburger Str. 45, 10117 Berlin, Germany; 5 Chair of Carbon Composites, TUM School of Engineering and Design, Technical University of Munich, Boltzmannstr. 15, 85748 Garching, Germany

**Keywords:** carbon fiber production, environmental impacts, life cycle assessment (LCA), life cycle inventory (LCI) data, energy analysis, scenario analysis

## Abstract

Carbon fibers (CFs) are a crucial material for lightweight structures with advanced mechanical performance. However, there is still a paucity of detailed understanding regarding the environmental impacts of production. Previously, mostly singled-out scenarios for CF production have been assessed, often based on scarce transparent inventory data. To expand the current knowledge and create a robust database for future evaluation, a life cycle assessment (LCA) was carried out. To this end, a detailed industry-approved LCI is published, which also proved plausible against the literature. Subsequently, based on a global scenario representing the market averages for precursor and CF production, the most relevant contributors to climate change (EF3.1 climate change, total) and the depletion of fossil energy carriers (EF3.1 resource use, fossil) were identified. The energy consumption in CF manufacturing was found to be responsible for 59% of the climate change and 48% of the fossil resource use. To enable a differentiated discussion of manufacturing locations and process energy consumption, 24 distinct scenarios were assessed. The findings demonstrate the significant dependence of the results on the scenarios’ boundary conditions: climate change ranges from 13.0 to 34.1 kg CO_2_ eq./kg CF and resource use from 262.3 to 497.9 MJ/kg CF. Through the investigated scenarios, the relevant reduction potentials were identified. The presented results help close an existing data gap for high-quality, regionalized, and technology-specific LCA results for the production of CF.

## 1. Introduction and Motivation

Lightweight structures are regarded as innovation drivers for sustainable and resource-saving businesses and make an important contribution to achieving climate protection goals. Research and development activities on the one hand focus on significant mass reduction, achievable either by using less material or by replacing it with lighter ones. On the other hand, enhanced functionality through intelligent and adaptive structures is often highly desired. In this context, carbon fibers (CFs) and the composites comprising them have been the subject of numerous research and development activities throughout various industrial sectors within the last few decades, as they are considered to provide advanced mechanical performance at a low weight and are therefore suitable for exploiting lightweight potentials [1,2,3]. Besides their mechanical performance, the main characteristics of carbon fibers are their high carbon contents of more than 95%, first-rate electrical conductivity, and physiological harmlessness [3].

The main applications for carbon fibers can be found in the aerospace industry, where carbon fiber-reinforced materials are crucial due to the significant fuel savings associated with their weight reduction potentials [4]. Carbon fiber-reinforced polymers (CFRPs) are also used, for example, as belt reinforcements in wind turbines. Due to the increasing expansion of wind energy, especially offshore, this sector now represents the second largest demand [4]. Further applications of carbon fibers can be found in small series sports cars [5], in mechanical engineering applications [6] as well as sporting goods production [7,8]. In the future, demand may particularly increase further in the area of hydrogen tanks in the transport sector [9,10], for off-shore wind turbines [4], and in the construction industry [11,12], mainly as reinforcement for concrete [13,14]. The existing potentials and advancements of carbon fiber technologies have been explored exhaustively in various scientific publications [11,12,15,16,17,18,19,20,21,22,23,24,25,26,27,28,29,30,31]. Particularly, in view of the historic and further increasing market relevance [4,32], the significant importance of carbon fibers is presumed to remain unchanged.

Despite the advantages mentioned above and numerous use cases, processing is complex and the environmental impacts of the production phase cannot be neglected [1]. Generally, these additional environmental expenses during manufacturing compared to other materials need to be compensated by either reduced material demand or savings during the use phase [33], which seems highly attractive, particularly for applications in the mobility sector, as in this case, large shares of resource consumption take place during the use phase [34]. The benefits of CF applications in aerospace are currently indisputable due to a dominating use phase [35,36]; however, automotive applications show a variegated pattern [37,38,39]. And, considering the ongoing energy transition away from fossil energy carriers, the use phase can be expected to become significantly greener in the coming years, even in the aviation industry. This decreasing influence of the use phase on the overall environmental performance of carbon fibers over the entire life cycle can also be expected for other fields of application. Consequently, the relevance of the manufacturing phase will progressively increase.

Notwithstanding the importance of reliable environmental sustainability assessments for CF manufacturing, so far, publicly available life cycle assessment (LCA) data on the production of CFs and CFRPs are often incomplete and not particularly transparent, even though carbon fibers are of significant interest for various applications [39]. This is mostly due to singular evaluations, which often neglect the influence different parameters such as energy consumption or production location can exert.

A detailed understanding of the environmental impacts of CF is, however, highly important; for example, when considering the overall environmental impacts of CFRP products. Depending on the production technology, amount of cuttings, matrix material, and other parameters, CFs were previously found to have a relevant influence on the environmental impact of resulting CFRP products [18]. The impacts amount to 43–84% of the climate change for fiber and their cuttings within processing [39].

Thus, this contribution aims to tackle the apparent gap in the research regarding the environmental sustainability of CF production under varying boundary conditions. Hereby, the primary research subject is a plausible life cycle inventory (LCI) for high tensile strength, high-tenacity fibers (HT-fibers) of average technical quality made from polyacrylonitrile (PAN), which make up the most significant proportion of the overall produced carbon fibers [3,30,40,41,42,43]. The basic LCI model for the production of CF is based on primary data; its outcomes have been internally reviewed and quality-assured by an industrial advisory board [18,39]. Subsequently, a life cycle impact assessment (LCA) according to the ISO 14040 series is carried out to quantify the environmental impacts of several scenarios for carbon fiber manufacturing. With this approach, instead of focusing on singled out results that only reflect one individual production plant with its inherent specifications, a bandwidth of potential results is presented. This is particularly crucial, considering historically grown equipment and production plants.

In conclusion, this paper contributes to the existing knowledge by putting together the current state of knowledge, providing an integrated summary of carbon fiber manufacturing, and deriving a dependable, industry-approved LCI, before calculating transparent and reliable LCIA results. In doing so, an initial evaluation is used to derive relevant scenarios for a subsequent in-depth investigation. The results of all the scenarios are summarized and discussed with regard to the data quality and possible fluctuations as well as further reduction potentials with regard to the environmental footprint. Consequently, with this paper, an existing gap within LCA can systematically be closed and a new understanding of the environmental impacts of carbon fibers is provided.

## 2. Overview of the Applied Methods

To quantify the environmental impacts of the production of carbon fibers, a life cycle assessment (LCA) is applied. It is a widely used method for the assessment of products, technologies, and processes along their entire life cycle regarding environmental implications [44]. The methodology is internationally standardized in ISO 14040 [45] and ISO 14044 [46] and is conducted iteratively in four phases: (1) goal and scope definition, (2) data collection in a so-called life cycle inventory (LCI), (3) deriving a quantification for the effects on the environment in the life cycle impact assessment (LCIA), and lastly, (4) the interpretation of the obtained results.

During this investigation, an extensive literature survey on the available LCI and LCIA data was carried out. The sources include different production locations, disregarding the intended applications of the produced carbon fibers. As variations between the reported values are evident, scientific papers and industrial publications were both considered to increase the overall data quality.

For the preliminary work [18,39], industrial data were collected that fundamentally describe the production of carbon fibers in terms of the mass and energy balance. Various model variants were derived from this basic model. Its outcomes have been internally reviewed and quality-assured by an industrial advisory board. To represent the worldwide production of carbon fibers as comprehensively as possible, this LCI model was built up in several variants. The investigations specifically include the following:

- The general process of CF manufacturing;
- The quantitative mass and energy flows of CF production;
- The influence of the energy sources in CF production;
- The environmental impacts of CF manufacturing;
- The most relevant countries for the production of CF;
- The identification of relevant levers to reduce overall environmental impacts of CF.

From the industrial LCI data, a model was derived, and our own calculations of the emission flows were integrated before the outcomes were quality-assured with the industrial primary data. For the required material and energy flows (such as nitrogen, water, etc.), background data from LCA for Experts CUP2023.1 were used [47]. Starting from that, an LCIA for CF manufacturing according to the international norms ISO 14040 and ISO 14044 was performed, considering a wide range of varying boundary conditions, which are presented at the end of Section 3. The calculation of environmental impacts follows the methodology of the environmental footprint (EF3.1) published by the European commission [48].

## 3. Description and Analysis of CF Production

According to Fitzer et al., there are generally two types of carbon fiber grades based on the same fundamental production process. Low-tow, ranging from 1 to 24 k filaments, and heavy-tow fibers [3]. Further differentiation can be made according to the final heat treatment [32], as well as the tensile elastic modulus and tensile strength [49]. Most of the produced carbon fibers are standard elastic modulus type (HT), which will consequently form the center of this investigation. All the energy flows discussed in the subsequent chapters are based on the average data for 12 k and 24 k roving. Using the same oven size or width, it is assumed that for 50 k fibers, the energy demand is still in a similar range, albeit a little lower.

### 3.1. Manufacturing Process

The principal production of CFs has been illustrated by various authors before. This article mainly follows the process description of the Japan Carbon Fibre Manufactures Association (JMCA) [50], the carbon fiber manufacturer Zoltek [51], and the manufacturing technology supplier Eisenmann [52,53] as well as the more detailed work about carbon fibers by Fitzer et al. [3], Griffing et al. [2,54], Morgan [55], Park [56], and Wetjen [30].

**Figure 1:**
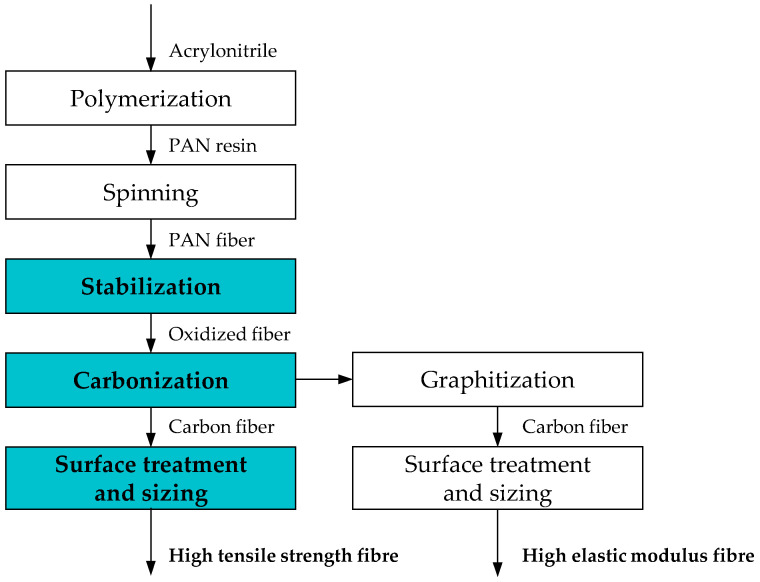
Carbon fiber manufacturing process from acrylonitrile based on [50,52]; the process steps for the production of high tensile strength carbon fibers from PAN are highlighted in blue.

With a significant share (more than 90%) of today’s high-performance CFs being made from polyacrylonitrile (PAN) [3,25,51], the paper at hand focuses on this precursor material and the corresponding processes illustrated in Figure 1. In order to improve the processability of PAN, a proportion of 2 to 5% of co-monomers such as vinyl esters and carboxylic acids is added during the production of PAN fibers [15], based on [49].

Generally, the investigated process of CF manufacturing can be described as a multi-stage chemical conversion of PAN fibers at elevated temperatures and subsequent surface treatment according to the depicted steps. Depending on the desired product, additional heating, also called graphitization, will result in fibers with an even higher modulus [32,50]. However, since this type of fiber only covers a marginal market share [3], it will not be considered further in this paper. As the environmental impacts of the precursor PAN fiber itself have already been investigated exhaustively and are available in LCA datasets [47], this paper will emphasize the subsequent stages. The scope of this report (highlighted blue in Figure 1) can generally be divided into three steps.

#### 3.1.1. Stabilization

Firstly, the fibers are stabilized by oxidation to preserve the original shape and avoid undesired thermal decomposition during carbonization [3]. To achieve this, in most industrial applications, the precursor is cautiously exposed to temperatures around 200 to 300 °C under air in a continuous oxidizing furnace for one to two hours while being under constant tension [3,25,50,55]. During the entire process, temperature and ventilation management is crucial, as stabilization comprises highly exothermic reactions severely affecting the fiber quality [51,53]. Since stabilization is one of the longest process steps in carbon fiber production, there are many research activities ongoing to increase productivity and reduce energy consumption. These include, for example, energy-efficient oxidation by means of plasma or microwave [57,58], waste heat recovery and utilization by preheating the incoming air through waste heat [59] as well as more efficient oven technologies using low-pressure stabilization and more precise temperature profile control [60], which results in a lower air flow and less process times. Stabilization is currently most often the focus of research activities since it takes significantly longer than the other process steps, and research activities focus primarily on increasing the productivity and shortening the cycle times.

#### 3.1.2. Carbonization

During the subsequent carbonization, fibers develop their superior mechanical properties [53]. The process is typically designed in two steps aiming to remove non-carbon atoms from the molecules within the fiber [55]. The first stage is performed at about 1000 °C in a low-temperature furnace made of stainless steel [53], also called a “tar removal furnace” [55]. Subsequently, the fibers are heated up until they reach 1500 to 1800 °C in an ensuing high-temperature graphite furnace [3,53]. The required high temperatures level out the differences in the processing times (carbonization requires a much shorter processing time than stabilization [3]). During the entire process, the fiber needs to be under continuous longitudinal tension to maintain its shape [50]. Additionally, to inhibit the further oxidation of the molecules, the high temperature furnace is operated under inert atmosphere, with nitrogen being the preferred inert gas [55]. Similar to the stabilization, more efficient heating technologies and the use of a heat recovery system have the potential to drastically reduce the energy consumption [41].

#### 3.1.3. Surface Treatment and Sizing

In the final step, the carbon fiber surface is treated, as the product of the carbonization does not bond well with the complementary composite material otherwise [30,51,55,56]. Some authors have considered the processing steps of the surface activation and application of the sizing agent individually [40,54]. During surface activation, the oxidization of the fiber surface is achieved through immersion in gaseous or liquid oxidants [51]. This prepares the fiber for the succeeding application of the sizing agent and particularly increases the wettability properties [30].

On an industrial scale, mainly electrochemical oxidation in an aquatic electrolyte solution is used due to its small and simple technology and low waste generation [30,56]. Hereby, the carbon fiber roving serves as the anode while being pulled through the electrolyte solution [54,55]. Subsequently, technical drying is performed [54]. Due to the predominance of the technology, this paper will focus on electrochemical surface treatment. Overall, the process control is critical in this step to avoid weakening the fiber by creating microscopic surface defects [51].

After the surface treatment, a washing process is integrated to clean the fiber [30]. Subsequently, a sizing agent is applied in order to protect the fiber in the following process steps from mechanical damage [54], and optimize the wettability with the matrix and fiber matrix bonding [34]. In industrial applications, coated carbon fibers are exclusively used [30,56]. Therefore, the sizing agent, also called the coating, is a polymer system with good chemical compatibility with the resin system used later [56]. Following Wetjen [30], epoxy coatings are applied in this study, since epoxy resins are assumed to be used. Nowadays, most of the sizing agents are water-based due to environmental and health issues caused by solvent-based sizing agents [55]. For sizing, the fiber is drawn through a sizing bath in which the sizing agent and water is absorbed. The water then evaporates during technical drying, is recovered, and fed back into the cycle, while the coating remains on the fiber [61].

#### 3.1.4. Supplementary Processes for Exhaust Gas Treatment

Both stabilization and carbonization generate emissions, where a significant amount of the PAN input mass is lost due to the removal of non-carbon atoms [54,55]. The occurring gaseous emissions must be treated and filtered before the waste air can be released into the environment, owing to the environmental and health implications. Typically, the thermal energy from natural gas combustion is used for the post treatment to achieve the necessary temperatures. On the other hand, this also means that the processes currently cannot be electrified completely, as gas is always required to support firing.

### 3.2. Quantification of Relevant Process Flows

**Figure 2:**
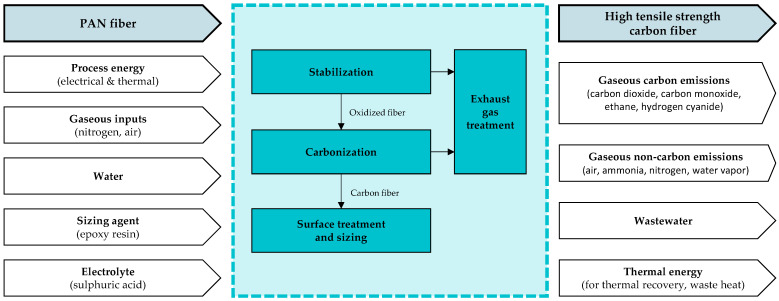
Schematic illustration of input and output flows of carbon fiber production from PAN and the required process steps considered in the scope of this paper; grey arrows: precursor and product, white arrows: auxiliaries and emissions, blue box: process steps.

The entire manufacturing process from PAN fibers to CFs needs various inputs and cause-related outputs. These need to be quantified for calculating the environmental impacts of carbon fiber production. The identified process flows are visualized in Figure 2. In the scope of this paper, all environmental impacts for the production of 1 kg of CFs are considered in a cradle-to-gate approach including precursors, energy demand, operating materials, waste, and emissions.

Besides PAN precursor fibers, educts include the process energy (electrical and thermal), additives, and processing agents in gaseous or liquid state, in particular, water, sizing agents, and electrolytes. While carbon fibers are the desired product, the processing also causes wastewater and gaseous emissions. Besides that, thermal energy occurs, which can partly be recovered; the rest is released into the environment, unused. Transport processes for precursor materials are not included in the defined system boundaries and need to be evaluated separately.

To quantify the output flows, an understanding of the chemical conversion process within the fiber material is vital. From PAN to PAN-based carbon fibers, the constituents carbon, nitrogen, hydrogen, and oxygen decrease significantly to reach a maximum carbon content of more than 95%wt. in the final product (c.f. Appendix A). The elimination of hydrogen and nitrogen atoms from the fiber leads to mass loss from the PAN input material. Various authors have provided quantification for this mass loss between 40 and 55 wt.% (c.f. Appendix A). According to Stiller, a majority of studies assume the weight loss exceeds 50 wt.% [62]. However, more recent work seems to indicate slightly lower values (c.f. Appendix A).

For the calculation of the environmental impacts, particularly, gaseous emissions need to be understood in detail. This is more challenging than the quantification of the input flows, as it is not immediately obvious which chemical compounds are released. Emissions have been discussed by various authors before, as presented in Appendix A. Since some of the gases originating from the stabilization and carbonization potentially cause significant environmental impacts, waste air purification in the manufacturing process is indispensable. A common practice is treatment by proprietary regenerative thermal oxidizers, offering more than 95% efficiency [55].

A second very relevant aspect for the environmental impacts of carbon fiber production is the required energy demand for stabilization and carbonization, which can differ due to various factors. On the one hand, carbon fiber plants are individually manufactured special plants that are in operation for decades. Depending on the production site, supply via gas or electricity is preferred, which, in turn, have different degrees of efficiency and associated environmental impacts. In summary, the age of the plant, the special plant itself, and the type of energy supply have an influence on the amount of energy required. So far, values were published that originate from calculations, expert interviews with carbon fiber manufacturers, or from the measurement of laboratory plants. This leads to a high range of published energy consumption values [61]. As already mentioned, the published values were checked and corroborated via an industry advisory board and a plausible average value for the total energy demand for the production of 12 k and 24 k HT-fibers was determined to be 170 MJ of thermal and electric per kg carbon fiber. As mentioned above, some of the thermal energy in the processes can be recovered; the rest is released into the environment. For an in-depth view, all input flows and their respective function within the processes, as well as the output originating from them, are discussed exhaustively in [61]. Therefore, it will not be repeated here. For the conducted LCA in the scope of this study, all data have been verified by an industrial advisory board [18,39]. In addition, the results were found to be in good agreement with scientific publications and publications by industry associations [63]. The life cycle inventory used for all models in the scope of this manuscript is displayed in Table 1.

## 4. Initial Hotspot Assessment

**Figure 3:**
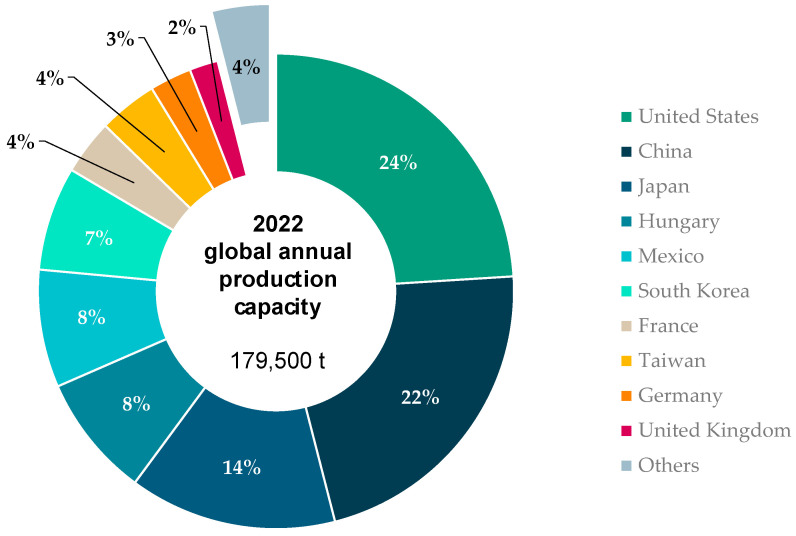
Global carbon fiber production capacity (2022) in the most relevant countries and regions, based on [4].

For an initial investigation of the hotspots and relevant levers for the environmental impacts of carbon fiber production, the presented LCI inventory (cf. Table 1) is used. Manufacturing takes place in various geographical regions, mostly in Asia, North America, and Europe, as reported in CU’s composite market report [4]. The numbers for the global production capacities in combination with the existing LCA background databases form the basis for the LCA conducted on a global production mix (cf. Figure 3). For the production of PAN fibers as the precursor, manufacturing in Japan is assumed. This assumption seems probable based on the fact that the Asia–Pacific region is the most relevant provider of all produced PAN worldwide (forecast 2023: 63% of global production) [64].

**Figure 4:**
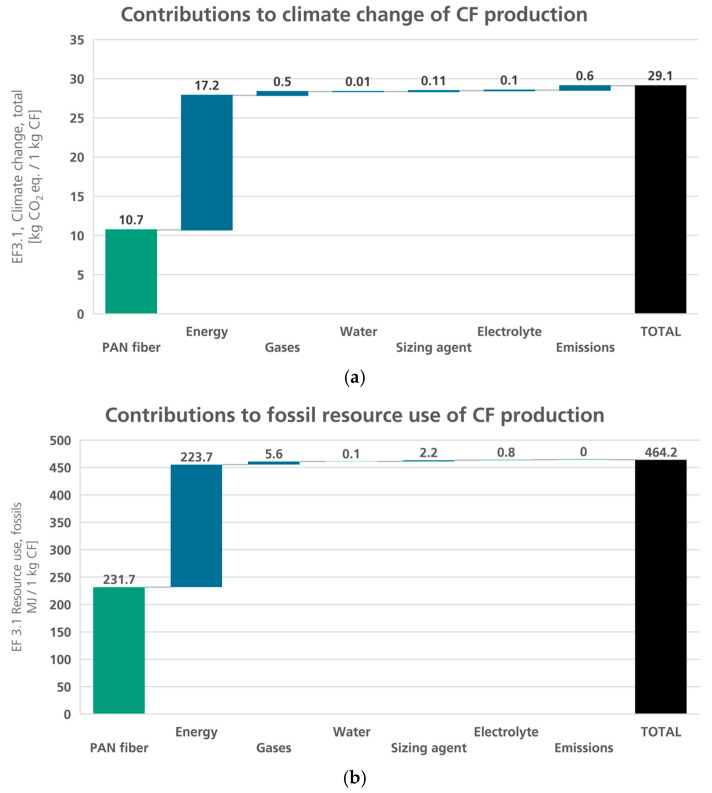
Results for the initial life cycle impact assessment of CF production regarding climate change (**a**) and resource use (**b**); green: precursor, black: product, blue: auxiliaries and emissions.

Based on the presented LCI inventory (cf. Table 1) and the global market mix for carbon fiber production capacities (cf. Figure 3), a life cycle impact assessment (LCIA) was conducted. The results were calculated using the harmonized impact categories of the Environmental Footprint EF3.1. Due to their relevance, the categories climate change, total as a parameter for the emission of greenhouse gases into the atmosphere and resource use, fossil as a metric for the depletion of non-renewable energy carriers were analyzed in this publication. Figure 4 breaks down the environmental impacts for the production of 1 kg of carbon fibers into PAN fibers, the energy (thermal and electric, including recovered thermal energy), gases (air and nitrogen), water, sizing agent, electrolyte, and emissions (gaseous carbon and non-carbon emissions and wastewater).

From this, the main contributors regarding climate change can be derived, as shown in Figure 4a. It is evident that both the manufacturing of the PAN precursor (37%) as well as the consumption of thermal and electrical energy (59%) are decisive. For the fossil resource use (c.f. Figure 4b), the trend is similar with slightly different shares: PAN fiber is responsible for 50%, and energy consumption for 48% of the resource use. Within the production of the PAN fibers themselves, again, the precursor material is the most relevant; in this case, the acrylonitrile (ACN) monomer, with shares of 76% of the climate change and 83% of the fossil resource use. The impact of the ACN production will not be altered for the scenarios discussed in this manuscript.

## 5. Investigated Variants and Scenarios

From the initial hotspot analysis, the relevant focus points for further investigation can be derived: the energy consumption during CF production and the provision of PAN fibers as precursor materials. Replacing PAN as the precursor material is not within the scope of this study. This leaves the energy sources for precursor and CF production, the production location, and the plant-specific technological setup as the relevant drivers for the environmental impacts of CFs. Therefore, in the development of the scenarios, the following aspects will be altered:

- Energy source precursor

For the energy source of the precursor material PAN, production in Japan is assumed analogously in the hotspot analysis. For the development of the scenarios, the energy source is varied between national Japanese grid mix and electricity from renewable energies. Japanese hydropower is selected as a representation of renewable electricity sources due to its currently high share in the Japanese renewable mix [65].

- Energy source CF production

The share of renewable energy sources is equally relevant for the carbon fiber production itself. Again, hydropower is selected as the representation of renewable electricity sources, as it is currently responsible for more than 50% of the total renewable world electricity generation in 2022, according to IEA [66]. Besides this aspect, which is applied to the global production mix as presented before, different geographical locations of production sites are equally relevant. For this purpose, individual country-specific scenarios reflect the use of different national grid mixes and electricity from national renewable sources. To this end, the most relevant countries in the grid mix are matched with existing LCA background data to result in the following scenarios: US, JP, CN, HU, DE, FR, and GB. The energy source for thermal energy is not varied across the different scenarios.

- Technologically optimized process (−50% energy demand)

With different measures, the energy consumption of fiber production can directly be influenced by the carbon fiber manufacturers. These include technological improvements like more efficient heating technologies and the use of a heat recovery system to increase the energy efficiency, as described for stabilization before. To show the potentials of technological optimization, an average energy reduction potential of stabilization and carbonization of 50% was assumed according to the research activities mentioned in Section 3.

For the global scenarios as well as for every country, three scenarios are modelled in the LCA for Experts software version 10.7.1.28 with the CUP 2023.1 database [47]. There are two scenarios with an average energy consumption, reflecting the technological state of the art. Both differ in the energy source for precursor production and CF production: the energy source is varied between the respective national grid mix or the national generation of hydropower as a representative of renewable energies. The third scenario considers the ongoing research activities to reduce energy consumption in stabilization and carbonization. An overview of all the evaluated variants and scenarios is given in Table 2.

## 6. Results of the Investigated Scenarios

**Figure 5:**
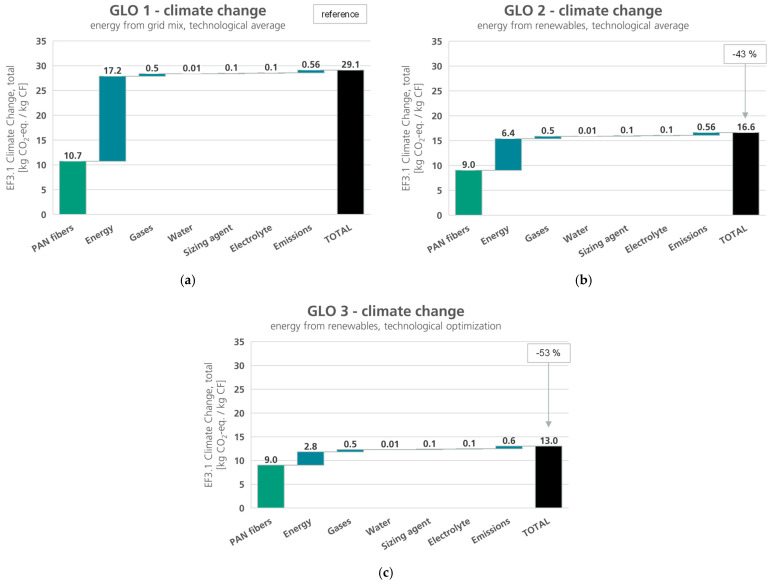
Climate change, global for carbon fiber production scenarios, and respective reduction potentials: (**a**) GLO 1—energy from grid mix, technological state of the art (c.f. Figure 4); (**b**) GLO 2—energy from renewables for PAN production and CF production, technological state of the art; (**c**) GLO 3—energy from renewables for PAN production and CF production, technological optimization; green: precursor, black: product, blue: auxiliaries and emissions.

**Figure 6:**
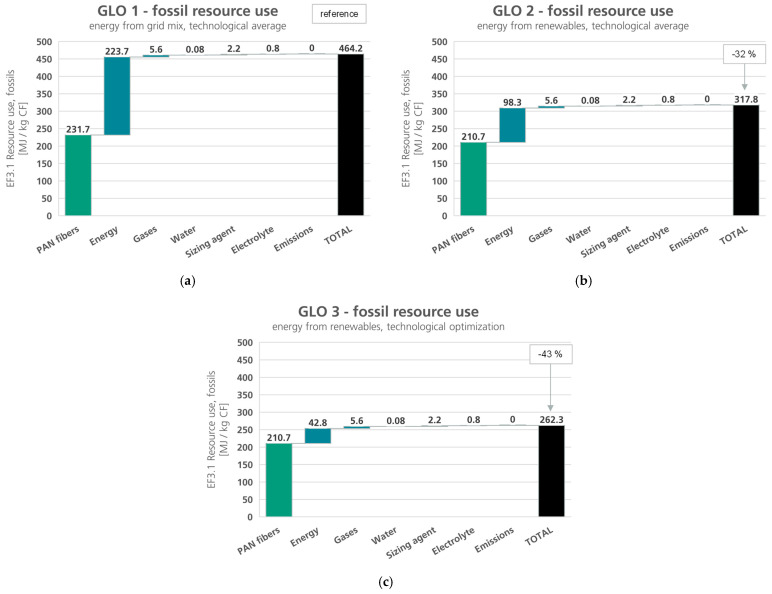
Fossil resource use, global carbon fiber production scenarios, and respective reduction potentials: (**a**) GLO 1—energy from grid mix, technological state of the art (c.f. Figure 4); (**b**) GLO 2—energy from renewables for PAN production and CF production, technological state of the art; (**c**) GLO 3—energy from renewables for PAN production and CF production, technological optimization; green: precursor, black: product, blue: auxiliaries and emissions.

In this section, the LCIA results for the scenarios described in the previous section are presented. The results of the global scenarios for the impact categories EF3.1 climate change, total and EF3.1 resource use, fossils are shown in Figure 5 and Figure 6. In both impact categories, the environmental impacts decrease from scenario GLO 1 to GLO 3, as to be expected. The environmental impacts caused by the provision of epoxy resin, nitrogen, and water, as well as the production of ammonium carbonate and the production of the carbon fiber do not change in the different scenarios.

The differences in the environmental impacts consequently arise solely from the use of electrical and thermal energy and the provision of the PAN fibers. Through a technology-optimized process and the use of renewable energies, a saving of approx. 53% in the climate change impacts, and a reduction of approximately 43% of the fossil resource use can be achieved in the best-case scenario (GLO 3).

**Figure 7:**
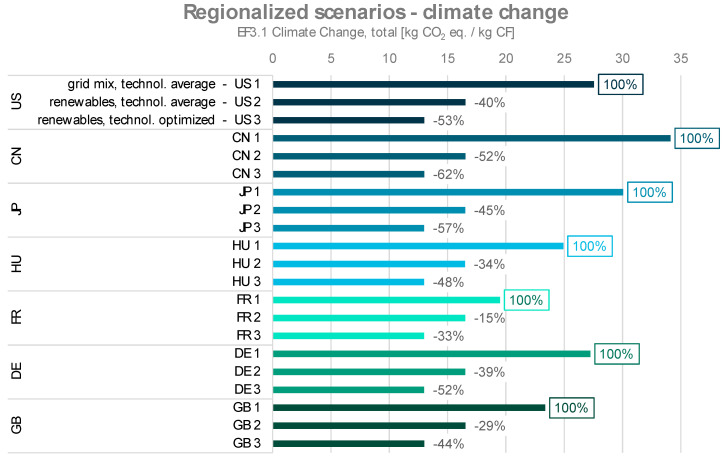
Climate change impacts of regionalized scenarios for carbon fiber production with the respective reduction potentials through energy from renewables (scenarios xx 2) and energy from renewables combined with technologically optimized process (scenarios xx 3).

**Figure 8:**
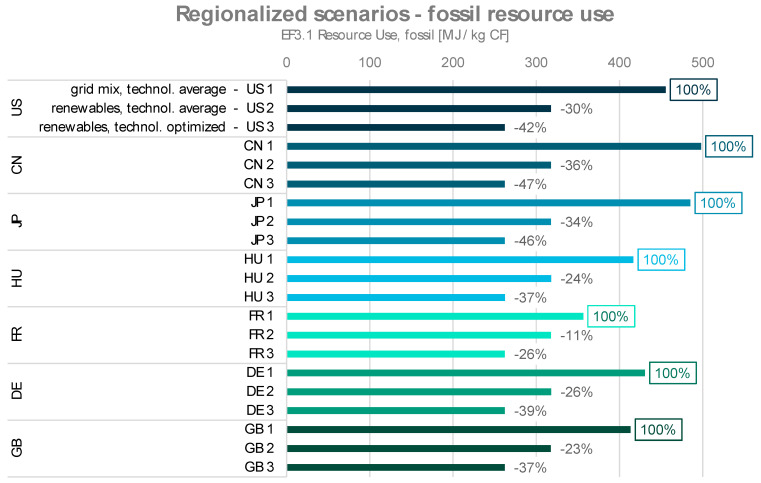
Fossil resource use impacts of regionalized scenarios for carbon fiber production with the respective reduction potentials through energy from renewables (scenarios xx 2) and energy from renewables combined with technologically optimized process (scenarios xx 3).

The results of the regionalized scenarios are shown in Figure 7 and Figure 8. Each region’s dataset consists of three scenarios, as described above. In both impact categories, for each region, the scenario “energy from grid mix, technological average” displays the highest environmental impacts, and the scenario “energy from renewables, technological optimization” shows the lowest.

When comparing the individual regions, it is noticeable that the environmental impacts for the optimized energy consumption and the use of hydropower differ only slightly between the regions. This is due to the already low environmental footprint for the procurement of 1 kWh of electricity from hydropower. Therefore, even reducing the energy consumption (and thereby their resulting environmental impacts) by 50%, as applied in the technologically optimized scenario, only results in a relatively small change. The datasets with the use of the regionalized grid mix differ more clearly. The reason for this lies in the environmental impacts of the grid mixes of the different regions from the applied background LCA database.

## 7. Discussion of the Results

The results of this study have demonstrated the significant variability in the calculated environmental impacts of carbon fiber production, depending on the selected boundary conditions. Even though obtaining data from literature is always subject to uncertainties, the collected inputs from a large number of authors offered a detailed overview of the bandwidth in which the life cycle inventory (LCI) values for the production of PAN-based carbon fibers lie. This is important, as carbon fiber plants themselves have grown historically and are complex specialized equipment. Thus, they also carry relevant uncertainties for the quantification of environmental impacts and there is never just one value when quantifying the mass and energy flows of the plants due to their technological variation.

The case-studies for individual plants are not the focus of this paper, yet an exhaustive, parameterized LCA model was developed that allows for the depiction of variations in detail or using the default market mix if the exact production parameters are unknown. In this case, the energy grid mix is deducted from the national grid mixes of CF-producing countries multiplied by their respective global market shares. Our own calculations and the industrial review were used to validate all the assumptions and literature values, and to ensure the establishment of a high-quality average life cycle inventory dataset.

Since there are still many parameters affecting the reliability of the LCIA results for CF manufacturing, it must be noted that site-specific primary data will always lead to more reliable and precise life cycle impact assessments of products than generic datasets. Even more so, CF plants are highly individualized special plants which can be in operation for decades. This is a challenge of particular interest for product-specific assessments of environmental sustainability, such as environmental product declarations (EPDs), or GHG scope 3 assessments. Moreover, uncertainties of CF manufacturing will propagate throughout the assessment of more complex CF-reinforced composites, making accurate data even more crucial.

While the focus on PAN-based carbon fibers (CFs) limits the scope of this work and does not allow for detailed statements about other precursor materials, pitch- and rayon-based CFs only play a minor role on the global market, as mentioned before [3,32,42,51], and their processing paths are technologically similar to those of PAN-based CFs [56]. Therefore, the investigation covers almost the entire market and allows for robust estimations, even for alternative precursor fibers. In the future, additional data for other precursor materials could be added to facilitate the comparison of this variable. Previous research by Hohmann presented possible pathways for a greener PAN fiber, based on Bio-Naphtha, Bio- or E-Methanol, or lignocellulose sugars [67].

Further variations in the environmental impacts of PAN should also be investigated, as its high relevance for the overall environmental impacts of CF production is evident. If additional scenarios for the value chain are implemented, such as different production scenarios for ACN, such as the precursor material to the CF-precursor PAN, it quickly results in a massive number of scenarios. This is equally true for the downstream value chain after CF manufacturing, as there is a plethora of different processing technologies resulting in specialized CRFP products.

In this case, modelling by hand in standard LCA software might no longer be the best choice and (semi-)automated approaches for LCIA calculations, as illustrated for extensive product portfolios before [68,69], should be considered. On the one hand, these approaches require adaptation to the specific challenges in carbon fiber production. On the other hand, the effort seems reasonable, as they can extend the subject of this study significantly, and thus create an even more complete understanding of CF value chains and their environmental impacts.

With the currently conducted scenario analyses, the results obtained from the life cycle impact assessment are comparable to those reported in the literature before [1,20,22,23,24,26,37,38,39,40,54,62,70,71,72,73,74,75,76,77,78,79,80,81,82]. However, the presented model offers significantly more modelling freedom and adaptability. The selectable settings include the following:

- Location-specific electricity grid profiles;
- The use of renewable energy sources for production;
- Accounting for new research results like technology-optimized processes in fiber production, resulting in a more efficient energy consumption.

The variation caused by these settings in the overall environmental footprint of PAN-based carbon fibers have been presented in detail in this manuscript. As mentioned several times, there is not one single value for the environmental impacts of carbon fiber production of a certain technical quality, as different processing parameters can lead to the same technical product with different associated environmental impacts from production.

The contribution to climate change for the global scenarios GLO 1 to GLO 3 range between 13 and 29 kg CO_2_ eq./kg CF. Only the Chinese scenario CN 1 (energy from national grid mix, no technological optimization) results in a higher value of 34.1 kg CO_2_ eq./kg CF. For this regionalized example, the shift from national electricity grid mix to solely renewable energy sources in combination with technological optimization can reduce the contribution to climate change for CF manufacturing from PAN by 62%. Unsurprisingly, the observed effect is biggest for countries like China, where the national grid mix is currently based mostly on coal power. The effect can, however, be observed for each scenario group and amounts to an at least 33% reduction potential (FR 3)

For fossil resource use, the results for the global scenarios GLO 1 to GLO 3 range from 262.3 to 464.2 MJ/kg CF. Here, the regionalized models for China (497.9 MJ/kg CF) and Japan (485.1 MJ/kg CF) result in higher values than the global scenarios. Again, France has the smallest lever for improvement within the investigated scenarios due to their relatively low CO_2_ footprint for electricity provision. Even in this case, the reduction potential is up to 26% compared to scenario FR 1.

In each set of the investigated scenarios, the third scenario (renewables for PAN production, renewables for CF production, optimized technology) can be interpreted to reflect a future in which the transition to renewable energy sources for industry and energy-efficient processing has been fully achieved. Yet, without addressing PAN production in further detail, the environmental impacts of CF manufacturing eventually meet a limit, below which they cannot be lowered through process optimization alone. The wide range of possible outcomes proves that even only fine-tuning the production process can already have a significant impact on the environmental performance. Consequently, the implementation of a large set of parameterized process conditions is critical for hotspot analysis on a manufacturer level. At the same time, the developed models can facilitate a general modelling of carbon fibers from PAN with default input data according to industry standards and average parameters. The respective reduction potentials for renewable energy sources in combination with a technological optimization for the regionalized models range from 26 and 47%.

At this point, it is pivotal to add that the country-specific evaluation should not carelessly be used as the only quantitative indicator for procurement decisions and the selection of suppliers. The presented values are intended to demonstrate the variability of the environmental impacts of carbon fiber production. Ideally, for procurement decisions, individual plant-specific data is to be used to account for sometimes significant variation between plats in one country and their individual energy sources. Moreover, the evaluation is based on current grid mixes, whereas procurement decisions often have long-term effects that stretch far beyond the temporal scope of this study. Lastly, to form a more complete basis for procurement decisions, the system boundaries must also be expanded to include transport for precursors and from the manufacturer to the customer.

As was pointed out in the introduction to this paper, CFs contribute a significant share to the environmental impacts of CFRP products. It is evident that the quantification of this contribution is highly dependent on the specific production scenario for CFs. The scenarios based on technological average and national grid mix presented in this study are in line with more than 50% of the climate change impacts of CFRPs according to [18]. Thus, achieving CF production with optimized CO_2_ footprints would reduce their contribution within the CFRP products and increase the relevance of other aspects. Consequently, the selection of the right LCIA for CFs is crucial and the use of arbitrary values from the literature will not suffice when modelling CFRP products. The authors therefore encourage further investigations of these aspects.

## 8. Conclusions and Recommendations

Even though carbon fibers are increasingly used in a multitude of different applications throughout many industrial sectors, their environmental performance still has proven difficult to quantify. Various authors have put forward calculations for the life cycle impacts of carbon fiber (CF) production, in which most of them are cross-referencing each other while sometimes producing conflicting numbers at the same time. The most relevant shortcoming of previous research is the singular and often untransparent evaluations of one specific technological setting and the lack of industry-approved inventory data. In this context, the presented work produced several key advances to the current understanding of the environmental impacts of CF production:

1. A scrutinized life cycle inventory (LCI) for the cradle-to-gate production of high-tenacity carbon fibers of average technical quality has been presented, which was approved by an industrial advisory board. The LCI also proved valid against various authors in the literature review. This baseline enables other researchers in the future to model CF production themselves, e.g., for the evaluation of additional impact categories or regional scopes, or for plant-specific assessments.
2. Building on the transparent LCI, an approved and consistent life cycle impact assessment (LCIA) following the ISO 14040 series was produced for a global “standard fiber” (cradle-to-gate) based on global market shares and technological averages for production. A substantial proportion of the overall impacts regarding climate change, total and resource use, fossil (acc. to EF3.1) result from the direct energy consumption in CF production (59% of climate change and 48% of the resource use, respectively), as well as from the manufacturing of PAN fibers as the standard precursor material (37 and 50%).
3. Lastly, different scenarios have been evaluated to create insights into potential levers for reducing environmental impacts. To this end, the energy source (for PAN fibers and for CF production), as well as a different plant condition (technological average vs. technological optimization) were considered. These variations were applied to the global CF market mix, as well as seven highly relevant supplier countries of CFs. Depending on the scenario and impact category, the environmental impacts of CF production could be reduced by between 11 and 62% at different geographical locations. The deviation between the results and reduction potentials highlights the dependency of the results on the scenario’s specific system boundaries and the investigated setup.

In conclusion, for any further life cycle study of CF-based product systems, the dependency of the LCA results on the initial assumptions, e.g., for energy provision, must be considered. To facilitate this, this work put forward a critically scrutinized LCI and an overview of the results for the assessment of a wide set of scenarios with a high degree of adaptability. All of this leads to a detailed overview and a solid, reliable baseline for future environmental considerations regarding the production of carbon fibers and composites comprising them. This can be used, for example to build more precise models representing case study-specific boundary conditions in the future. The authors particularly want to encourage further involvement with two vital aspects of sustainability of carbon fibers: a material-based optimization of precursor sustainability and the transfer of findings from this work into further life cycle assessments on the application of CFs in CFRPs.

## Figures and Tables

**Table 1:** Quantitative life cycle inventory (LCI) for the production of one kilogram of carbon fibers (CFs) from polyacrylonitrile (PAN), as used in the models in the scope of this manuscript.

	Flow	Amount	Unit
**Input**	Polyacrylonitrile (PAN) fiber	1.95	[kg/kg CF]
	Air	9.6	[kg/kg CF]
	Nitrogen [N_2_]	10.0	[kg/kg CF]
	Electrolyte [Ammonium bicarbonate]	0.03	[kg/kg CF]
	Sizing agent [Epoxy resin]	0.02	[kg/kg CF]
	Water [H_2_O]	5.5	[kg/kg CF]
	Process energy (electric and thermal)	170	[MJ/kg CF]
**Output**	**Carbon fiber**	**1.00**	**[kg]**
	Gaseous carbon emissions [HCN, C_2_H_6_, CO_2_, CO]	0.6	[kg/kg CF]
	Gaseous non-carbon emissions [exhaust air, N_2_, NH_3_, H_2_O (g)]	20.0	[kg/kg CF]
	Wastewater	5.5	[kg/kg CF]
	Recovered thermal energy	11.2	[MJ/kg CF]
	Waste heat	8.32	[MJ/kg CF]

**Table 2:** Overview of all investigated variants and scenarios for global and regionalized production of carbon fibers under different boundary conditions.

Variant	Scenario	Energy Source Precursor Production	Energy Source CF Production	Technologically Optimized Process (−50% Energy Demand)
Global	GLO 1	Grid mix (JP)	Grid mix (GLO)	No
	GLO 2	Hydropower (JP)	Hydropower (GLO)	No
	GLO 3	Hydropower (JP)	Hydropower (GLO)	Yes
US	US 1	Grid mix (JP)	Grid mix (US)	No
	US 2	Hydropower (JP)	Hydropower (US)	No
	US 3	Hydropower (JP)	Hydropower (US)	Yes
Japan	JP 1	Grid mix (JP)	Grid mix (JP)	No
	JP 2	Hydropower (JP)	Hydropower (JP)	No
	JP 3	Hydropower (JP)	Hydropower (JP)	Yes
China	CN 1	Grid mix (JP)	Grid mix (CN)	No
	CN 2	Hydropower (JP)	Hydropower (CN)	No
	CN 3	Hydropower (JP)	Hydropower (CN)	Yes
Hungary	HU 1	Grid mix (JP)	Grid mix (HU)	No
	HU 2	Hydropower (JP)	Hydropower (HU)	No
	HU 3	Hydropower (JP)	Hydropower (HU)	Yes
Germany	DE 1	Grid mix (JP)	Grid mix (DE)	No
	DE 2	Hydropower (JP)	Hydropower (DE)	No
	DE 3	Hydropower (JP)	Hydropower (DE)	Yes
France	FR 1	Grid mix (JP)	Grid mix (FR)	No
	FR 2	Hydropower (JP)	Hydropower (FR)	No
	FR 3	Hydropower (JP)	Hydropower (FR)	Yes
GB	GB 1	Grid mix (JP)	Grid mix (GB)	No
	GB 2	Hydropower (JP)	Hydropower (GB)	No
	GB 3	Hydropower (JP)	Hydropower (GB)	Yes

## Data Availability

All relevant LCI data are included in the article and its appendix, further input data used for the LCIA calculations are available to users of LCA for Experts software. Any additional data can be shared upon request with permission of all involved authors.

## Appendix A. Background Data for Emission Modelling

To understand the inputs and outputs of carbon fiber production and derive a robust life cycle inventory, understanding the emissions plays a pivotal role. The change in the chemical composition of fibers from PAN to CFs is the baseline for this (c.f. Table A1).

**Table A1:** Chemical composition of the fiber at subsequent processing steps along the process chain of carbon fiber (CF) manufacturing from polyacrylonitrile (PAN) according to Fitzer et al. [3].

	Constituents [wt.%]			
	C	N	H	O
PAN fiber	68	26	6	-
Stabilized PAN fiber	65	12	5	8
Carbonized PAN fiber (<500 °C)	67	19	11	3
Carbonized PAN fiber (<700 °C)	72	18	7	2
PAN-based carbon fiber (~1500 °C)	>95	4.5	0.3	0.2

The mass share of carbon is increasing in the process, whereas the overall fiber mass decreases through the gaseous emission of the different components. This mass loss has been reported by various authors as between 41 and 55%, as shown in Table A2.

**Table A2:** Overview of literature values for the total mass loss in the production of 1 kg carbon fibers (CF) from polyacrylonitrile (PAN).

Literature Source	Required Amount of PAN Fiber for the Production of 1 kg of CF [kg]	Corresponding Loss of PAN Fiber during Production Process [wt.%]
[83]	~2	~50% **
[2,54]	1.82 **	45% *
[62] based on [84]	2 to 2.2 **	50 to 55% *
[70] based on [85]	1.82 to 2 **	45 to 50% *
Supplementary data for [24]	1.75	43% **
[26] based on [54]	1.82	45% **
[40]	1.695	41% **
[1]	2.08 **	52% *
[3]	1.82 to 2 **	45 to 50% *
[77] based on [54]	1.82	45% **
[86]	1.67 to 2 **	40 to 50% *

* Calculated from CF yield after oxidization and carbonization, ** calculated from the respective corresponding value given in study.

For modelling the emissions resulting from fiber stabilization and carbonization, the mass loss is assumed to be roughly 49%, which equals the needs of 1.95 kg PAN fiber to produce 1 kg of carbon fiber. This mass loss translates into the respective gaseous emissions, for which different chemical compounds have been reported qualitatively by others before, as summarized in Table A3. A quantitative emission profile has been put forward by Schmidt and Watson, based on Griffing and Overcash, as listed in Table A4.

**Table A3:** Qualitative overview of emissions from stabilization and carbonization in CF manufacturing, as reported by various sources (X indicates if source has mentioned the respective emission).

Source	HCN	NH_3_	NO_X_	H_2_O	CO	CO_2_	H_2_	N_2_	VOCs	HCs (C_2_H_4_, C_2_H_6_)	CH_4_	Other
[3]	X	X	X						X			
[51]		X		X	X	X	X	X				X
[54]	X	X		X	X	X	X	X		X		
[86] based on [87]	X	X		X	X	X	X	X			X	

**Table A4:** Emissions from stabilization and carbonization in CF manufacturing (per produced kilogram of CF), as reported by Schmidt and Watson [77] based on Griffing and Overcash [54].

Emission		Mass	Emission Path
Sulfuric acid	H_2_SO_4_	0.0199 kg/kg CF	water
Ethane	C_2_H_6_	0.0000101 kg/kg CF	air
Ammonia	NH_3_	0.00116 kg/kg CF	air
Hydrogen cyanide	HCN	0.0157 kg/kg CF	air
Carbon monoxide	CO	0.00324 kg/kg CF	air
Carbon dioxide	CO_2_	1.013 kg/kg CF	air

As mentioned in the article, waste air purification in the manufacturing process is indispensable. A common practice is treatment by proprietary regenerative thermal oxidizers, currently already offering more than 95% efficiency. Table A5 lists the efficiency factors for the thermal treatment of waste gases in CF manufacturing, as reported by Morgan et al. [55].

**Table A5:** Efficiency factors for the thermal treatment of different compounds in the exhaust gas from stabilization and carbonization, as reported by Morgan et al. [55].

Emission		During Stabilization	During Carbonization
Ammonia	NH_3_	95 to 99 wt.%	95 to 99 wt.%
Hydrogen cyanide	HCN	95 to 99 wt.%	95 to 99 wt.%
Ethane	C_2_H_6_	-	95 to 99 wt.%
Ethene	C_2_H_4_	-	100 wt.%
Carbon monoxide	CO	95 to 99 wt.%	95 to 99 wt.%
Methane	CH_4_	-	100 wt.%
Hydrogen	H_2_	-	100 wt.%

For the chemical conversion during the exhaust gas treatment, the following ideal chemical equations are used. It should be noted that during the combustion of hydrogen cyanide and ammonia, depending on the oxygen present, residence time, and combustion temperature, nitrogen, nitrogen monoxide, or nitrogen dioxide may be produced [88]. All the presented reactions are exothermal (${\Delta H}_{R}<0$).

**Table A6:** Ideal chemical equations for the thermal treatment of exhaust gas during carbon fiber manufacturing and standard enthalpies of reaction (${\Delta H}_{R}^{{}^{\circ}}$).

Combustion of ammonia		
$\;4{NH}_{3}(g)+7O_{2}(g)⇌4NO_{2}\left(g\right)+6H_{2}O(l)$	${\Delta H}_{R}^{{}^{\circ}}$ ≈ −1399.6 kJ/mol	(A1)
$\;4{NH}_{3}\left(g\right)+5O_{2}\left(g\right)⇌4NO\left(g\right)+6H_{2}O(l)$	${\Delta H}_{R}^{{}^{\circ}}$ ≈ −1170.8 kJ/mol	(A2)
$\;4{NH}_{3}(g)+3O_{2}\left(g\right)⇌2N_{2}\left(g\right)+6H_{2}O(l)$	${\Delta H}_{R}^{{}^{\circ}}$ ≈ −1532.0 kJ/mol	(A3)
Combustion of hydrogen cyanide		
$\;4HCN+9O_{2}(g)⇌4CO_{2}+4NO_{2}+2H_{2}O(l)$	${\Delta H}_{R}^{{}^{\circ}}$ ≈ −2556.2 kJ/mol	(A4)
$\;4HCN+7O_{2}(g)⇌4CO_{2}+4NO+2H_{2}O(l)$	${\Delta H}_{R}^{{}^{\circ}}$ ≈ −2327.4 kJ/mol	(A5)
$\;4HCN+5O_{2}\left(g\right)⇌4CO_{2}+2N_{2}+2H_{2}O(l)$	${\Delta H}_{R}^{{}^{\circ}}$ ≈ −2688.6 kJ/mol	(A6)
Combustion of ethane		
$\;2C_{2}H_{6}\left(g\right)+7O_{2}\left(g\right)⇌4CO_{2}\left(g\right)+6H_{2}O(l)$	${\Delta H}_{R}^{{}^{\circ}}$ ≈ −3124.0 kJ/mol	(A7)
Combustion of ethene		
$\;C_{2}H_{4}(g)+3O_{2}(g)⇌2CO_{2}\left(g\right)+2H_{2}O(l)$	${\Delta H}_{R}^{{}^{\circ}}$ ≈ −1412.0 kJ/mol	(A8)
Combustion of methane		
$\;CH_{4}\left(g\right)+2O_{2}(g)⇌CO_{2}(g)+2H_{2}O(l)$	${\Delta H}_{R}^{{}^{\circ}}$ ≈ −816.0 kJ/mol	(A9)
Combustion of carbon monoxide		
$\;2CO\left(g\right)+O_{2}(g)⇌2CO_{2}(g)$	${\Delta H}_{R}^{{}^{\circ}}$ ≈ −566.4 kJ/mol	(A10)
Combustion of hydrogen		
$\;2H_{2}\left(g\right)+O_{2}(g)⇌2H_{2}O(l)$	${\Delta H}_{R}^{{}^{\circ}}$ ≈ −572.0 kJ/mol	(A11)

Note: values for ${\Delta H}_{R}^{{}^{\circ}}$ are calculated from standard enthalpies of formation (${\Delta H}_{f}^{{}^{\circ}}$; based on NIST Chemistry WebBook [89]) and rounded to one digit. Due to uncertainties in values for ${\Delta H}_{f}^{{}^{\circ}}$, other literature might report slightly different numbers.

In contrast to Khalil [20], the final combustion calculation is included after the determination of these gases, during which the efficiency factors listed in Table A5 are taken into account for the thermal treatment of exhaust air. Finally, for the applied life cycle inventory (cf. Table 1 in the main text of this article), the detailed input flows for all further materials as well as the energy input flows are quantified using primary data that are verified by an industrial advisory board [18,39]. The emission flows are supplemented by our own calculations, based on [61].
